# Liver transplant and hepatitis C in methadone maintenance therapy: a case report

**DOI:** 10.1186/1747-597X-2-5

**Published:** 2007-02-01

**Authors:** Meredith M Hancock, Colette C Prosser, Kanat Ransibrahmanakul, Laura Lester, Elana Craemer, James A Bourgeois, Lorenzo Rossaro

**Affiliations:** 1 Section of Transplant Medicine, Department of Internal Medicine, University of California Davis Medical Center, 4150 V Street, PSSB, Suite 3500, Sacramento, CA 95817, USA; 2 Division of Gastroenterology and Hepatology, Department of Internal Medicine, University of California Davis Medical Center, 4150 V Street, PSSB, Suite 3500, Sacramento, CA 95817, USA; 3 Department of Psychiatry and Behavioral Sciences, University of California Davis Medical Center, 2230 Stockton Blvd., Sacramento, CA 95817, USA

## Abstract

Methadone maintenance therapy for the treatment of opioid dependence continues to carry a social stigma. Until recently, patients on methadone were not considered for liver transplantation. We describe the first case of a patient on methadone who received a liver transplant for end stage liver disease and was successfully treated for recurrent hepatitis C. More than five years post transplant and three years post viral clearance, the patient continues to do well and is stable on low-dose methadone. This case emphasizes the need to reconsider the non-evidence based policy adopted by transplant centers that require methadone maintenance therapy patients to stop methadone prior to consideration for transplant evaluation.

## Background

Although there are years of data proving methadone as a highly effective treatment for opioid dependence, methadone maintenance therapy (MMT) continues to be a barrier to standard medical care. Even today, there is a general misconception that MMT patients make poor candidates for liver transplantation (LT) despite the lack of evidence that their overall outcome differs from non-MMT patients [1,2]. In 2000, a nationwide survey showed that only 56% of responding transplant centers (90% of the queried) considered active MMT patients for transplant waiting lists; however, 32% of respondents required patients to cease methadone prior to transplantation [3].

In our transplant center at UC Davis Medical Center (UCDMC), patients referred for liver transplant undergo standard psycho-social, financial, medical, and surgical evaluations. Patients who meet the criteria for substance dependence sign an abstinence agreement which may require toxicology screenings and documented attendance to Narcotics/Alcoholics Anonymous meetings (NA and AA, respectively). Patients on methadone are not excluded or required to cease MMT, although they may be expected to attend NA. The duration and consistency of attendance to the MMT program, including routine toxicology screenings and counseling, are considered by the LT selection committee on a case by case basis. UCDMC does not operate its own MMT program, thus patients must obtain this service elsewhere.

HCV is highly prevalent in MMT patients[4]. Although a recent study showed that MMT patients did have a slightly decreased sustained virological response (SVR) to antiviral therapy with interferon (IFN) and ribavirin (RBV) (28%), it clearly showed that treatment is possible [5]. The disparity in response rates can be attributed to many factors including smaller cohort size and selected patient population (clinical degree of liver disease, concurrent mental illness, and current or recent substance abuse).

Our case demonstrates that a patient on MMT can receive LT and successfully be treated for HCV recurrence post transplant.

## Case report

In August 2000, a 37 year old Native-American male received a cadaveric liver transplant after approximately 20 years of chronic HCV infection, genotype 1a. He was a former intravenous drug user (IVDU) and had been enrolled in a MMT program since 1990, taking between 80 and 100 mg methadone daily. He also had a history of anxiety and mood disorders, and had been taking clonazepam, 1 mg 4 times daily since 1999. At transplant evaluation, the patient had solid psychosocial support (family and counseling) and had a consistent ten year adherence to his MMT, including negative toxicological screens. The UCDMC transplant team determined that it would be in the patient's best interest to continue his MMT pre and post transplant.

The patient had a MELD score of 15 at the time of transplant. He tolerated the operation well with no major complications during surgery, although he did require more intraoperative anesthesia than the average non-MMT patient. Post-operative pain management during hospitalization required consultation with pain specialists and the doses of hydromorphone and morphine were higher than the average non -MMT transplant patients. On post-operative day six, the patient resumed a normal diet; his pain was managed with oral narcotic analgesics; he was fully ambulatory and was discharged from the hospital. Immediately post-transplant, the patient's methadone was 100 mg daily.

In June 2001, the patient showed biopsy proven recurrent HCV in the transplanted liver. There were no signs of rejection. The patient was continuing MMT ~80 mg daily and was compliant with his immunosuppressive therapy and transplant follow up care. In January 2002, the patient began HCV treatment using an escalating regimen of INF combined with RBV. He reached full dose INF (3 MIU three times per week) and RBV (1200 mg daily) at approximately 10 weeks of treatment. The intention was to treat for 48 weeks on combination therapy. The patient started treatment with a low viral load (410,760 IU/mL). At approximately six months of therapy, he was HCV-RNA quantitatively virus negative.

Overall, the patient had common side effects to the combination therapy. His physical symptoms included fatigue, nausea, headaches, and flu-like symptoms. These side effects were treated symptomatically and either resolved or were well-tolerated. Although the patient was taking immunosuppressive drugs throughout his HCV therapy, his white blood cell and absolute neutrophil counts remained stable. By treatment week 12, he did experience significant hemolytic anemia (Hgb 10.7 g/dL) and was started on 40,000 units per week of erythropoietin. The patient's RBV was reduced to 800 mg daily for two weeks and then was increased to 1000 mg daily for the remainder of treatment.

Due to a history of anxiety and mood disorders, the patient was started on low dose antidepressants, citalopram 10 mg daily, three months prior to starting treatment. Additionally, the patient experienced psychiatric side effects to IFN/RBV including mood swings, angry outbursts, insomnia, and forgetfulness. His citalopram, 10 mg daily, was increased twice (20 mg daily at week 12 and 40 mg daily at week 40). Quetiapine, 25 mg 1–2 tabs daily, and bupropion, 150 mg twice daily, were also added halfway through treatment to further control these psychiatric side effects. These additional psychotropic medications were managed in conjunction with the patient's methadone clinic; during treatment the patient's methadone was gradually increased from 84 mg daily to a maximum of 110 mg daily. Twenty-four weeks after treatment, the patient's methadone was reduced to 80 mg daily and has incrementally decreased to his current dose of 4 mg daily in 2006. The patient has continued to take clonazepam, 1 mg 3 times daily, and quetiapine, 50 mg daily, post treatment. In 2004, he was able to find a job after many years of unemployment. In 2006, four years after treatment, he continues to be: (a) HCV free with annual liver biopsies showing no signs of disease or fibrosis, (b) stable on immunosuppression, (c) on minimal doses of methadone (15 mg/d), and (d) compliant with medical advice and medications.

## Discussion

Stigma regarding methadone maintenance therapy continues to be a barrier to patients receiving liver transplant and standard HCV treatment. Although methadone is a widely-accepted, highly effective treatment for opiate addiction, MMT patients continue to be discriminated against. The primary reason for this bias was investigated in a 2000 survey which showed that most transplant centers required MMT cessation prior to LT. The general conclusion was that there was a significant misunderstanding between heroin dependency and methadone maintenance therapy as treatment[1]. More specifically, methadone is often seen as an abused drug, not as a treatment for opiate addiction. Thus, MMT patients may still be perceived as not fully "recovered"; they are treated as if they are "addicted" to a drug (i.e., methadone) and are not "worthy or ready" for standard medical care, such as treatment of HCV and LT. Many transplant centers require patients to discontinue MMT before they can be considered for transplant – a practice which may be considered unethical and harmful; however, there is an emerging opinion to support the cautious inclusion of MMT patient in liver transplantation [6,7].

The NIH 2002 Consensus Statement on HCV management clearly supports methadone treatment for opiate addiction. The document states that methadone helps reduce risky behaviors and should not be used as a reason to exclude a patient from HCV treatment. Additionally it affirms that methadone patients (and even active IVDUs and alcoholics) can be treated successfully for HCV [8]. Model MMT patients have a proven track record of medical follow up appointments, repeated laboratory studies, and adherence to taking their methadone. It has been suggested that patients enrolled in MMT are the best candidates for HCV therapy among IVDUs [9].

Opioid dependent patients may have pre-existing mood and/or anxiety disorders. In addition, these patients are at risk for IFN-associated mood disturbances. When these patients are treated for mood and/or anxiety disorders with antidepressants, physicians are advised to monitor doses, side effects, and drug-drug interactions closely and make medication changes as needed [10].

MMT patients who undergo LT require more intraoperative anesthesia and postoperative analgesia than non-MMT patients [11]. Our patient is no exception. However, this should not be used as an argument to deny LT for MMT patients.

Our case study demonstrates that a correctly evaluated patient on MMT can achieve SVR even after LT. Although, organ transplantation and decompensated cirrhosis are on the list of contraindications for use of IFN-based therapy, most transplant hepatologists are now treating both difficult to treat groups. It would be ideal for HCV patients to clear the virus before transplant since reinfection of the graft after liver transplant in patients who are HCV positive at the time of transplant is universal [12]. In addition, due to the donor shortage, many centers have adopted expanded donor policies that include transplanting HCV positive donors to HCV positive recipients. However, those rare patients who are able to clear the virus with therapy before liver transplant have a low rate of recurrence and should not receive HCV positive livers.

To our knowledge, this is the first report of successful treatment of HCV post-transplant in an MMT patient. We recommend that patients on MMT needing a liver transplant should not be required to stop taking methadone. We believe that each patient should be evaluated independently and equally as far as their history and compliance behavior. Furthermore, although HCV treatment may be more difficult in transplant patients on immunosuppression and methadone, it should be considered and tested in larger trials.

## Competing interests

The author(s) declare that they have no competing interests.

## Authors' contributions

MH participated in the data analysis and drafted the manuscript. CP participated in data collection and analysis, and completion of the manuscript. KR participated in the data analysis and completion of the manuscript, LL participated in the data analysis and completion of the manuscript, EC participated in the data analysis and helped to draft the manuscript, JB participated in data collection and analysis, and completion of the manuscript, LR conceived of the study, and participated in its design and coordination and helped to draft the manuscript. All authors read and approved the final manuscript.
